# From Eat to trEat: engineering the mitochondrial Eat1 enzyme for enhanced ethyl acetate production in *Escherichia coli*

**DOI:** 10.1186/s13068-020-01711-1

**Published:** 2020-04-19

**Authors:** Aleksander J. Kruis, Anna C. Bohnenkamp, Bram Nap, Jochem Nielsen, Astrid E. Mars, Rene H. Wijffels, John van der Oost, Servé W. M. Kengen, Ruud A. Weusthuis

**Affiliations:** 1 Laboratory of Microbiology, Wageningen University and Research, Stippeneng 4, 6708 WE Wageningen, The Netherlands; 2 Bioprocess Engineering, Wageningen University and Research, Droevendaalsesteeg 1, 6708 PB Wageningen, The Netherlands; 3 Biobased Products, Wageningen University and Research, Bornse Weilanden 9, 6708 WG Wageningen, The Netherlands; 4 Faculty of Biosciences and Aquaculture, Nord University, 8049 Bodø, Norway

**Keywords:** Eat1, Alcohol acetyl transferase (AAT), Mitochondria, *Escherichia coli*, Ethyl acetate

## Abstract

**Background:**

Genetic engineering of microorganisms has become a common practice to establish microbial cell factories for a wide range of compounds. Ethyl acetate is an industrial solvent that is used in several applications, mainly as a biodegradable organic solvent with low toxicity. While ethyl acetate is produced by several natural yeast species, the main mechanism of production has remained elusive until the discovery of Eat1 in *Wickerhamomyces anomalus*. Unlike other yeast alcohol acetyl transferases (AATs), Eat1 is located in the yeast mitochondria, suggesting that the coding sequence contains a mitochondrial pre-sequence. For expression in prokaryotic hosts such as *E. coli*, expression of heterologous proteins with eukaryotic signal sequences may not be optimal.

**Results:**

Unprocessed and synthetically truncated eat1 variants of *Kluyveromyces marxianus* and *Wickerhamomyces anomalus* have been compared in vitro regarding enzyme activity and stability. While the specific activity remained unaffected, half-life improved for several truncated variants. The same variants showed better performance regarding ethyl acetate production when expressed in *E. coli.*

**Conclusion:**

By analysing and predicting the N-terminal pre-sequences of different Eat1 proteins and systematically trimming them, the stability of the enzymes in vitro could be improved, leading to an overall improvement of in vivo ethyl acetate production in *E. coli*. Truncated variants of *eat1* could therefore benefit future engineering approaches towards efficient ethyl acetate production.

## Background

Ethyl acetate production in yeast is catalysed by alcohol acetyltransferases (AATs), which synthesise ethyl acetate from acetyl-CoA and ethanol, releasing free CoA [10]. The first described ethyl acetate-producing AAT was the *Saccharomyces cerevisiae* alcohol acetyltransferase 1 (Atf1) [17]. However, its homologs in *Wickerhamomyces anomalus* and *Kluyveromyces marxianus* appeared to have only a minor role in bulk ethyl acetate production [7, 14]. Instead, these microorganisms use the recently identified ethanol acetyltransferase (Eat1) to produce ethyl acetate. All ethyl acetate-producing yeasts were shown to possess at least one functional Eat1 homolog [7]. Besides AAT activity, Eat1 enzymes exhibit esterase and thioesterase activities as well, hydrolysing ethyl acetate and acetyl-CoA, respectively. The hydrolysing activities of *W. anomalus* Eat1 could be suppressed in vitro by sufficiently high levels of ethanol [7, 19].

A key difference between Atf1 and Eat1 is their cellular location in yeast. Atf1 localises to lipid particles in the cytosol [13, 27], while Eat1 homologs are located in yeast mitochondria [9, 15]. Most mitochondrial enzymes like Eat1 are encoded on the nuclear genome and synthesised in the cytoplasm. They are transported into the mitochondria via the translocase of the outer mitochondrial membrane (TOM) complex, based on the presence of mitochondrial targeting signals. These targeting sequences contain amphipathic helices, which partially destabilise the nascent proteins and facilitate the cross-membrane transport into the mitochondria [31]. Most mitochondrial proteins contain N-terminal pre-sequences that are cleaved by mitochondrial proteases, with mitochondrial processing peptidase (MPP) being the most prominent. In some cases, other peptidases like Oct1 or Icp55 initiate additional cleavage events of the pre-protein [18]. Icp55 cleaves one additional amino acid (AA) from MPP-generated N-termini, while Oct1 removes another 8 AA after cleavage by MPP or Icp55 [29].

Mitochondrial cleavage events of Eat1 in native yeasts have not been studied in much detail. In the *K. marxianus* Eat1, removal of the initial 19 AA prevented localisation to the native yeast mitochondria. It is unclear whether this is the final, mature form of Eat1, or if additional cleavage events occur after MPP cleavage [15]. Imo32, a *S. cerevisiae* homolog of Eat1 [7], is processed by both MPP and Oct1 [28]. It is possible that multiple processing events occur in other Eat homologs as well. However, the precise final form of Eat proteins can only be determined through experiments in the native hosts, such as isolation of mature Eat1 from yeast mitochondria. These procedures include isolation of intact yeast mitochondria through differential centrifugation, which is laborious. Furthermore, the purification of Eat1 would likely require an in vitro assay. To this point, the AAT activity of Eat1 in cell-free extracts has not been reported, which makes this approach difficult.

Ethyl acetate production by native yeasts on an industrial scale is limited by the yeast metabolism due to the specific environmental conditions that are required for ester synthesis. These include iron or oxygen limitation, which are difficult to control on an industrial scale [7, 9, 25]. Alternative hosts, especially bacteria and archaea could be used instead. However, prokaryotic hosts are unable to cleave mitochondrial pre-sequences, which may lead to hampered heterologous expression of *eat1* and impaired ethyl acetate production. Introduction of the mature, final forms of the mitochondrial Eat1 in prokaryotes would likely improve in vivo ethyl acetate production. In this study we improved ethyl acetate production in *Escherichia coli* by truncating the N-terminus of Eat1 enzymes from *W. anomalus* and *K. marxianus*. To determine the optimal truncation position, 16 Eat1 variants were produced in *E. coli*, and tested in vivo and in vitro for their effect on ethyl acetate production.

## Results

### In silico analysis of Eat1 N-terminal sequences

**Fig. 1:**
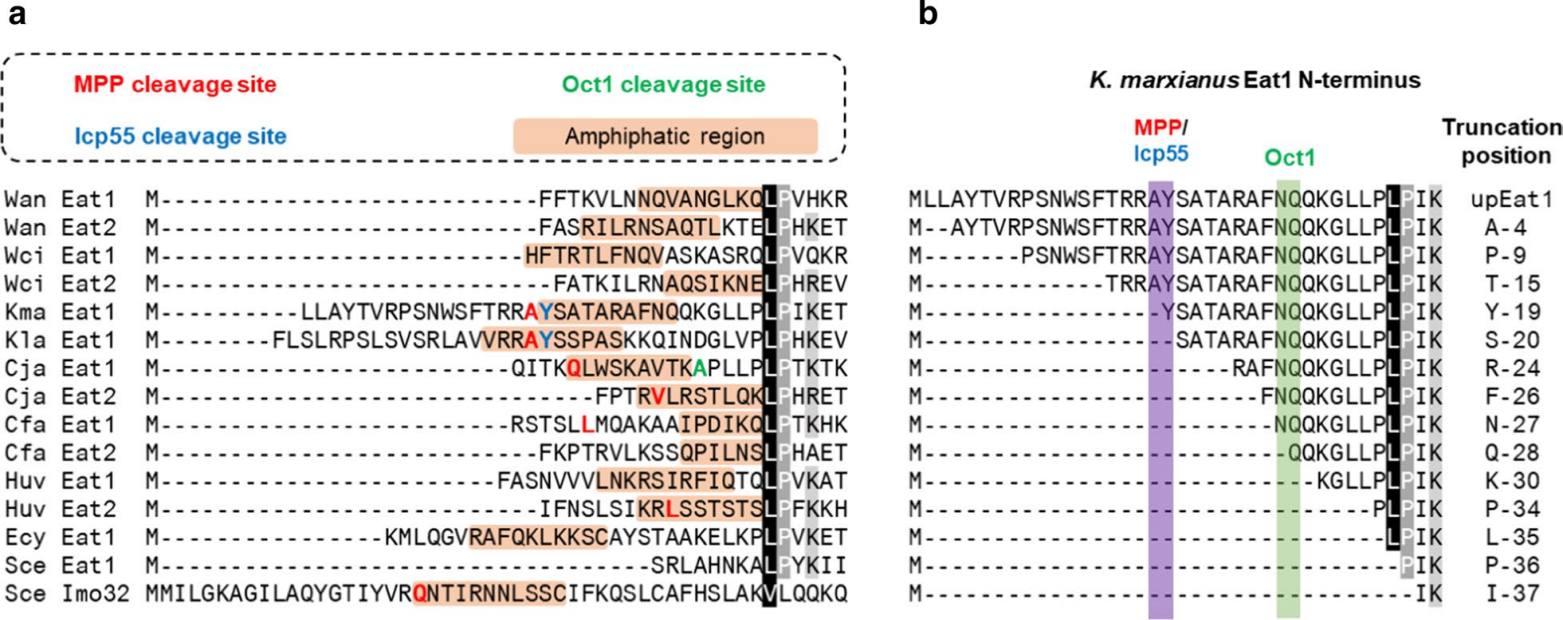
In silico analysis of 15 Eat homologs from various yeasts using MitoFates [4]. **a** Prediction of amphipathic regions and putative cleavage sites in Eat N-termini. **b** Design of 15 Kma trEat1 variants. Wan—*Wickerhamomyces anomalus*, Wci—*Wickerhamomyces ciferrii*, Kma—*Kluyveromyces marxianus*, Kla—*Kluyveromyces lactis*, Cja—*Cyberlindnera jadinii*, Cfa—*Cyberlindnera fabianii*, Huv—*Hanseniaspora uvarum*, Ecy—*Eremothecium cymbalariae,* Sce—*Saccharomyces cerevisiae*

Optimal function of Eat1 in *E. coli* likely depends on introducing the mature, truncated form of the enzyme. This form is not known for any of the Eat1 homologs that are able to produce ethyl acetate and can only be determined in the native yeast hosts through laborious experiments. Instead, we searched in silico for predicted cleavage sites within the N-termini of 15 Eat homologs from various yeast species using MitoFates [4]. All but the *S. cerevisiae* Eat1 N-termini contained an amphipathic region that is typically observed in N-terminal sequences of mitochondrial proteins (Fig. 1a). Several sequences also had predicted MPP/Icp55 cleavage sites. Curiously, the predicted MPP/Icp55 cleavage sites would not fully remove the destabilising amphipathic region of their respective Eat1 N-termini. Since the amphipathic regions destabilise protein folding [31], they are presumably removed during enzyme processing in the mitochondria. This may indicate that additional cleavage events, such as Oct1 cleavage in Cja Eat1 (Fig. 1a) occur in Eat1.

We focused on N-termini of the *Wickerhamomyces anomalus* (Wan) Eat1 and *Kluyveromyces marxianus* (Kma) Eat1. Both enzymes are derived from yeasts that are able to produce high amounts of ethyl acetate. Efficient ethyl acetate synthesis by unmodified (but codon-harmonised) Wan Eat1 has already been demonstrated in *E. coli* [7]. However, the composition of the Wan and Kma Eat1 N-termini is remarkably different. The longer Kma Eat1 contained a clear pre-sequence and recognition sites for two mitochondrial peptidases, MPP and Icp55 at amino acid (AA) positions 19 and 20, respectively (Fig. 1a). The shorter N-terminus of Wan still showed the characteristic amphipathic region, but no clear mitochondrial peptidase motifs were detected (Fig. 1a). We therefore initially focused on optimising the N-terminus of the Kma Eat1. We designed 14 truncated versions of Kma Eat1 (trEat1) based on predicted cleavage sites, as well as arbitrary positions within the N-terminus. The truncated variants are denoted by the first AA appearing after the cleavage position (Fig. 1b), although in reality, all proteins begin with M.

### Expression of truncated Kma Eat1 variants in *E. coli*

Ethyl acetate production from glucose by the truncated Kma Eat1 (Kma trEat1) variants was assessed in *E. coli.* The cells were grown under anaerobic conditions to stimulate production of ethanol, which is required by Eat1 to produce ethyl acetate. The carbon flux was channelled towards ethyl acetate production by disrupting the lactate dehydrogenase (ldhA) and acetate kinase (ackA) genes. This eliminated lactate production and lowered acetate formation, respectively (results not shown). The resulting *E. coli* BW25113 Δ*ackA*Δ*ldhA* (DE3) strain was used to express the *eat1* gene variants under the control of the LacI/*T7* promoter.

**Fig. 2:**
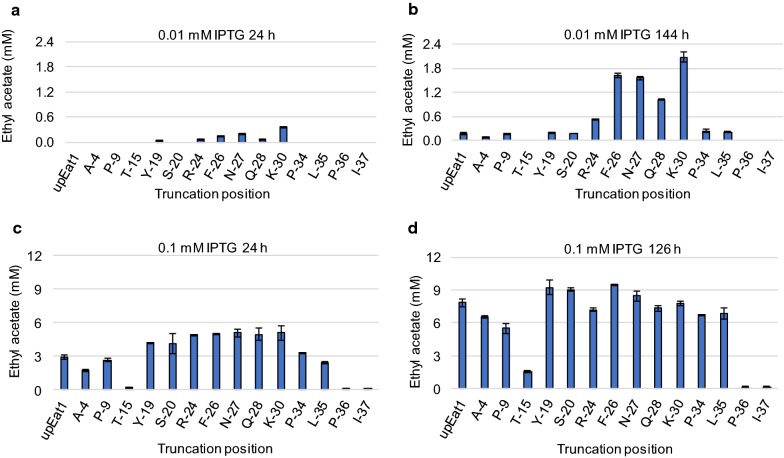
Improved ethyl acetate production by N-terminal truncated versions of Kma Eat1. **a**, **b** Ethyl acetate titres reached by cultures producing Kma trEat1 variants at 0.01 mM IPTG after a 24 h and b 144 h. **c**, **d** Ethyl acetate titres reached by cultures producing Kma trEat1 variants at 0.1 mM IPTG after c 24 h and d 126 h. Strains were grown under anaerobic conditions in modified M9 medium. Genes were expressed from a series of pET26b plasmids. Experiments were performed as biological duplicates; error bars represent the standard deviation. Abbreviations: Kma, *K. marxianus*

We induced gene expression with 0.01 mM IPTG and 0.1 mM IPTG (Fig. 2). At the lowest concentration, a profoundly positive effect on the final ethyl acetate titre was observed with several truncated variants compared to the untruncated (up) Eat1 (Fig. 2a, b). At 0.1 mM IPTG, the differences in ethyl acetate titres produced by Kma upEat1 and the Kma trEat1 variants were less apparent (Fig. 2c, d). Since 0.1 mM IPTG is a high inducer concentration, it is likely that ethyl acetate production was not limited by the AAT activity of Eat1, but instead by other metabolic bottlenecks. However, the low ethyl acetate production at 0.01 mM IPTG suggests that ethyl acetate production was limited by the activity of Kma Eat1. Any changes in the ethyl acetate production can therefore be linked directly to the in vivo activity of the enzymes.

The BW25113 Δ*ackA*Δ*ldhA* (DE3) strains producing Kma trEat1 F-26, N-27, Q-28 and K-30 all formed ethyl acetate within 24 h of cultivation, whereas no ethyl acetate was detected in the strains producing the unprocessed Kma Eat1 and most other Kma trEat1 variants (Fig. 2a). During the second time point (144 h) all Eat1 variants produced detectable amounts of ethyl acetate, except Kma trEat1 T-15, P-36 and I-37. Nevertheless, Kma trEat1 F-26, N-27, Q-28 and K-30 produced substantially more ethyl acetate compared to the unprocessed control and other Eat1 variants. The best performer was *E. coli* BW25113 Δ*ackA*Δ*ldhA* (DE3) producing Kma trEat1 K-30, which formed 11.8-fold more ethyl acetate than the unprocessed variant corresponding to 2.07 mM or 182.4 mg/L (Fig. 2b). *E. coli* BW25113 Δ*ackA*Δ*ldhA* (DE3) producing Kma trEat1 P-9, Y-19, S-20, P-34 and P-35 formed approximately the same amounts of ethyl acetate as the unprocessed Kma Eat1 which produced around 0.18 mM (15.5 mg/L) ethyl acetate (Fig. 2).

Most trEat1 variants either led to increased ethyl acetate production or did not affect it significantly (Fig. 2a, b). The exceptions were the strains producing Kma trEat1 T-15, P-36 and I-37, which formed only traces of ethyl acetate. The Kma trEat1 P-36, and I-37 removed the first conserved region that is present in all Eat1 homologs from various yeasts [7], which indicates that this conserved region is critical for ethyl acetate formation by Eat1. It is unclear why ethyl acetate formation was severely reduced in the strain producing Kma trEat1 T-15 (Fig. 2).

Improved in vivo performance of Kma trEat1 F-26 and K-30 may be linked to improved protein solubility. To test this, soluble and insoluble fractions of cell-free extracts (CFE) were prepared during ethyl acetate formation by unprocessed Kma Eat1 and trEat1 K-30 (Additional file 1: Figure S1a). Gene expression was induced with 0.01 mM IPTG. Most of the Kma Eat1 was in the insoluble fraction. Truncating the protein did not affect this, indicating that improved ethyl acetate production by Kma trEat1 K-30 was not caused by improved protein solubility.

An alternative explanation may be that truncating the 5′ coding sequence of Kma *eat1* affected the translation initiation rates of the ribosome binding sites (RBS) used for protein translation. To exclude this possibility, we calculated the translation initiation rates for each Kma *trEat1* gene using the RBS Calculator [22]. We compared the translation initiation rates with the ethyl acetate titres achieved by *E. coli* BW25113 Δ*ackA*Δ*ldhA* (DE3) producing the Kma trEat1 variants with 0.01 mM IPTG (Fig. 2a) and found little correlation between them (*r* = − 0.14, Additional file 2: Figure S2). This supports the hypothesis that truncating the N-terminus of Kma Eat1 affected its function on the protein level.

### Improved in vitro stability of Kma trEat1 variants

**Fig. 3:**
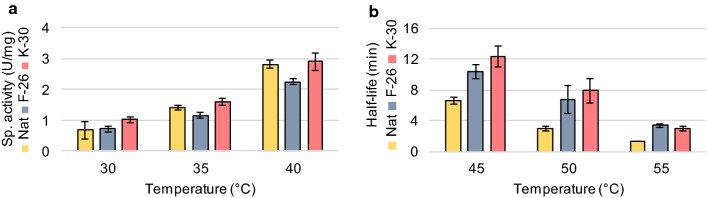
Improved stability of N-terminal truncated versions of Kma Eat1. **a** In vitro specific activity and **b** half-life of purified unprocessed Kma Eat1, Kma trEat1 F-26 and K-30 at various temperatures. Esterase activity was measured by following the release of 1-naphthol at 320 nm from 1-naphthyl acetate. Measurements were performed as technical triplicates; error bars represent the standard deviation

The improved ethyl acetate production was presumably caused by changes to Eat1 on the protein level, either by a higher specific activity or by an enhanced stability. To test this, we purified the unprocessed Kma Eat1, Kma trEat1 F-26, and Kma trEat1 K-30, and measured their initial 1-naphthyl acetate (1-NA) hydrolysis rates at 30 °C, 35 °C and 40 °C based on the esterase activity of Eat1 (Fig. 3a). Hydrolysis of 1-NA releases free 1-naphthol, which can be measured spectrophotometrically. The 1-NA assay was used in place of direct measurement of ethyl acetate synthesis (AAT activity) since the method is considerably less laborious and more sensitive. The specific esterase activities of the three proteins moderately increased with temperature, with a 10°K increase leading to a threefold higher specific activity (Fig. 3a). The truncated variants of Kma Eat1, however, did not exhibit a higher specific activity compared to unprocessed Eat1. We then tested whether truncating Kma Eat1 affected the stability of the proteins by determining their half-lives at 45 °C, 50 °C and 55 °C. For both Kma trEat1 F-26 and K-30, the half-lives were significantly higher at all tested temperatures compared to the unprocessed Kma Eat1 (Fig. 3b, Additional file 3: Figure S3). Kma trEat1 F-26 and K-30 were thus more thermostable. Apparently, the N-terminal region has a weakening effect on the thermostability of Eat1.

### Expression of truncated Wan Eat1 variants in E. coli

**Fig. 4:**
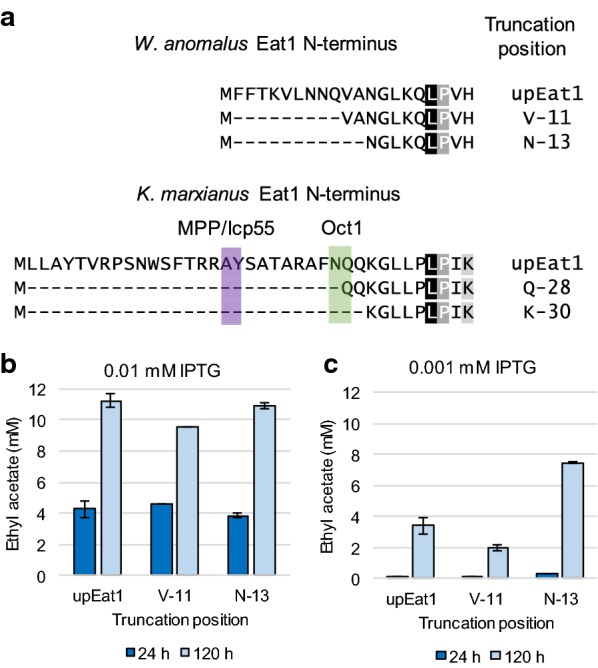
Improved ethyl acetate production by N-terminal truncated versions of Wan Eat1. **a** truncated variants of the Wan Eat1 N-terminus based on the Kma trEat1 Q-28 and K-30. The highlighted LP residues indicate the first region that is conserved in all known Eat1 proteins. **b** Ethyl acetate titres reached by cultures producing Wan trEat1 variants at 0.01 mM IPTG induction levels after 24 h (dark blue) and 120 h (light blue). **c** Ethyl acetate titres reached by cultures producing Wan trEat1 variants at 0.001 mM IPTG induction levels after 24 h (dark blue) and 120 h (light blue) Strains were grown under anaerobic conditions in modified M9 medium. Genes were expressed from a series of pET26b plasmids. Experiments were performed as biological duplicates; error bars represent the standard deviation. Wan—*W. anomalus*, upEat1—unprocessed Eat1

We examined whether the function of Wan Eat1 could also be improved by truncating its N-terminus. Predicting the structure of the N-terminal localisation sequence of Wan Eat1 using MitoFates did not result in clearly defined protease cleavage positions. Therefore, we used the conserved region at AA positions 36 and 37 within the Kma N-terminus as a guide to create two Wan trEat1 variants. Kma trEat1 Q-28 and K-30 were used to generate their Wan trEat1-V11 and N-13 counterparts, respectively (Fig. 4a). The variants were produced in *E. coli* BW25113 Δ*ackA*Δ*ldhA* (DE3) under the control of the LacI/*T7* promoter. Interestingly, ethyl acetate titres exceeded measured values of Kma Eat1 already after 24 h when induced with 0.01 mM IPTG. All three strains producing the Wan Eat1 variants formed approximately 4 mM ethyl acetate (352 mg/L), and no significant difference could be observed between unprocessed and truncated Eat1s (Fig. 4b). After 120 h of fermentation, ethyl acetate concentrations varied between 9 and 11 mM or 792 and 968 mg/L, which was higher than all values obtained with the Kma Eat1 variants at 0.1 mM IPTG (Figs. 2c, d, 3b). This suggests that 0.01 mM IPTG was sufficient to fully induce Wan *eat1* expression to a point where the activity of Eat1 did not limit ethyl acetate synthesis.

To more accurately study the effect of the truncations, we lowered the IPTG concentration to 0.001 mM IPTG (Fig. 4c). The ethyl acetate formation trends in *E. coli* BW25113 Δ*ackA*Δ*ldhA* (DE3) producing Wan trEat1 N-13 at 0.001 mM IPTG were similar to those observed in strains producing the Kma K-30 at 0.01 mM IPTG (Figs. 2a, b and 4c). At both sampling points, the strain producing Wan trEat1 N-13 reached a twofold higher ethyl acetate concentration than the unprocessed Wan Eat1 (Fig. 4b). No difference was found between the Wan trEat1 V-11 and the unprocessed Wan Eat1 after 24 h, while over a longer time period the truncated variant even produced less ethyl acetate than the other two tested strains (Fig. 4b, c). The CFE extracts of cultures producing unprocessed Wan Eat1 and Wan trEat1 N-13 were also analysed by SDS-PAGE during ethyl acetate formation (Additional file 1: Figure S1b). Similar to Kma Eat1, the overall solubility of Wan Eat1 did not improve after the protein was truncated.

## Discussion

We described the optimisation of functional expression of the mitochondrial Eat1 enzyme from yeasts in a prokaryotic host. The in vivo function of the mitochondrial Eat1 proteins in *E. coli* could be improved by removing the destabilising N-termini of the proteins. The Kma trEat1 F-26, N-27, Q-28 and K-30 contained potential cleavage sites that are located 7–11 AA residues after the predicted MPP cleavage site of Y-19 and S-20, indicating that one or more of them may be the mature form of Eat1. By removing the N-terminus, in vivo ethyl acetate production was improved as much as 11.8-fold in Kma trEat1 K-30 and twofold higher in Wan trEat1 N-13. Because the specific activity of the truncated versions was not significantly different from the non-truncated control, as shown for Kma variants, improved ethyl acetate production must have been caused by improved stability of the enzyme. This higher stability most likely leads to a higher number of active Eat1 proteins in vivo, causing the higher ethyl acetate titres. The prediction of the RBS strength of each individual truncated Kma trEat1 gene showed no correlation to the ethyl acetate titre, supporting this explanation.

While the cultures producing unprocessed Eat1 variants were analysed by SDS-PAGE, it became apparent that most of the enzyme was located in the insoluble fraction of the CFE. Truncation of Eat1 did not affect this distribution to a measurable extent. A similar trend was observed when *S. cerevisiae* Atf1 was expressed in *E. coli* and largely formed insoluble aggregates with reduced specific activity [32]. This suggests that a parts of the Eat1 as well as the trEat1 proteins remain unfunctional, which gives room for further improvement of ethyl acetate production by Eat1.

In yeast, the N-termini are removed by mitochondrial peptidases during protein translocation from the cytosol to the mitochondria, releasing the mature and stable protein [2, 29]. *E. coli* and other prokaryotic hosts cannot perform these processing events. Cleavage sites were only predicted within the N-terminus of Kma Eat1. The strains producing Kma trEat1 variants that were truncated at the predicted positions (Kma trEat1 Y-19 and S-20) did not show substantially improved ethyl acetate production relative to the unprocessed Kma Eat1. However, removing 19 AA from Kma Eat1 (Kma trEat1 S-20) was indeed sufficient to fully prevent Eat1 from being targeted to the mitochondria of *K. marxianus* [15]. The ethyl acetate production only improved when an additional 7–11 AA residues were removed from the N-terminus of Kma Eat1. These variants were the Kma trEat1 F-26, N-27, Q-28, and K-30. They were chosen based on the processing events that occur in the *S. cerevisiae* Imo32, which may be a distant homolog of the Kma Eat1 [7, 28]. This may suggest that similar events occur during the processing of Eat1 in *K. marxianus*. The fact that Kma trEat1 S-20 is unable to migrate to the mitochondria in *K. marxianus* [15], but did not show improved performance in *E. coli* supports this hypothesis. It is likely that Eat1 homologs from other yeasts undergo different processing events as well. For example, the N-terminus of Wan Eat1 had no predicted cleavage sites and is roughly half the length of its *K. marxianus* counterpart, while truncation improved ethyl acetate production in Wat Eat1 N-13. Confirming the true final forms of Eat1 proteins is only possible by analysing the proteins in the native yeasts and may help to identify the most functional trEat1 variants.

The strains producing Wan Eat1 variants consistently formed 10–15% more ethyl acetate in vivo compared to strains producing Kma Eat1. They also required about twofold lower induction levels to achieve this. It has been shown that inducer concentrations affect growth and impose an additional metabolic burden to the cell, next to plasmid maintenance [1, 16]. Lower induction levels are therefore more desirable and screening for optimum levels is strongly recommended.

In this study, up to 4 mM or 352 mg/L ethyl acetate was produced within 24 h of anaerobic cultivation. While cultivation conditions, cell densities and working volumes affect the final outcome, reported titres for ethyl acetate production in *E. coli* settled in the 20 mg/L range [12, 20]. In contrast, isobutyl acetate production by *E. coli* using the *S. cerevisiae* Atf1 reached 17-36 g/L [20, 24]. This may be related to a lower affinity of the AATs used in those studies with respect to ethanol or acetyl-CoA and supports choice of Eat1 for efficient ethyl acetate production in *E. coli.* Unprocessed Wan Eat1 was used in *E. coli* to produce 5 g/L ethyl acetate under aerobic condition and ethanol supplementation [7]. The truncated Eat1 variants have the potential to increase this further. Whether Eat1 can also be used to produce other esters in *E. coli* has not been confirmed. Given that Eat1 contributes to acetate ester in *S. cerevisiae* in general, this seems plausible [8].

The better performance of Wan Eat1 compared to Kma Eat1 may also originate from its temperature optimum. The yeast *W. anomalus* is routinely cultivated at temperatures between 25 and 30 °C [3, 11, 21, 23]. In contrast, the yeast *K. marxianus* produced ethyl acetate more efficiently at 42 °C compared to lower temperatures [25]. As we cultivated the *E. coli* BW25113 Δ*ackA*Δ*ldhA* (DE3) strains at 30 °C, it is possible that Kma Eat1 was less efficient in *E. coli* BW25113 Δ*ackA*Δ*ldhA* (DE3) due to suboptimal cultivation temperatures for the enzyme. Our enzyme assays showed that the specific activity of Kma Eat1 was much higher at 40 °C than at 30 °C, supporting this hypothesis. While at low induction levels truncated variants performed consistently better, the benefits were less prone in anaerobic fermentations at higher inducer concentrations. Further analysis of the Kma trEat1 F-26 and K-30 in vitro revealed that the specific activity of the enzyme was unaffected by the truncations. Instead, their stability was improved as shown by the increased half-life at all tested temperatures compared to the unprocessed variant. In vivo, this improved stability of the trEat1 variants is reflected by the earlier appearance of ethyl acetate synthesis during the fermentation, and the higher difference in ethyl acetate production at the lowest IPTG concentrations. The limited stability of the unprocessed Eat1 enzymes can probably be compensated by higher IPTG concentrations, as *E. coli* BW25113 Δ*ackA*Δ*ldhA* (DE3) producing the unprocessed Eat1 or the truncated trEat1 variants reached comparable ethyl acetate titres when induced with higher IPTG concentrations.

## Conclusion

Expression of heterologous genes generally requires further optimisation. By acknowledging the mitochondrial origin of Eat1, we improved stability and in vivo performance of the enzyme in prokaryotic cells.

Systematic trimming of the N-terminus was performed on *eat1* originating from *K. marxianus* and *W. anomalus* leading to truncated variants with enhanced performance. Cleavage events exceeding the pre-sequence on the other hand, led to the loss of activity, highlighting the importance of the conserved region for functionality of the proteins.

Ethyl acetate production by *E.* coli BW25113 Δ*ackA*Δ*ldhA* (DE3) was improved by 10-15% for variants, that were trimmed 7–11 AA residues next to the predicted MPP cleavage site. While in vitro enzyme activities remained unaffected, half-life of the truncated enzymes increased, indicating higher stability. Expression of variants Kma trEat1 F-26 and K-30, as well as the corresponding Wan trEat1 V-11 and N-13, resulted in highest ethyl acetate titres in vivo. Additionally, the level of induction could be reduced, compared to the unprocessed variants.

Removal of the mitochondrial pre-sequence therefore contributed to better in vivo performance of the Eat1 enzyme in *E. coli.* The results can benefit further engineering approaches using *E. coli* as expression system for the efficient production of ethyl acetate.

## Materials and methods

### Strain and plasmid construction

The strains and plasmids used in this study are shown in Tables 1 and 2, respectively. Gene disruptions were performed with CRISPR–Cas9 [6] using 50 bp homologous regions immediately upstream and downstream of the ATG and stop codon, respectively. The pTarget and pET26b plasmids were assembled using the 2X HiFi assembly master mix (NEB) according to supplier instructions. All *K. marxianus* and *W. anomalus eat1* genes were cloned with a Strep-tag or 6-His-tag, respectively, to facilitate protein purification. The pTarget sequences containing homologous regions and the gRNA module were ordered synthetically as gBlocks (IDT). PCR amplifications were performed with Q5 polymerase (NEB) according to supplier instructions. Plasmids carrying truncated versions of *eat1* genes were constructed by PCR-amplifying either pET26b-hKmaEat1 or pET26b-hWanEat1 with primers that excluded the appropriate part of the 5′ sequence of the *eat1* gene. The reverse primer included the ATG codon and was phosphorylated at the 5′ end. The linear PCR product was circularised using T4 ligase (Thermo Scientific) according to manufacturer instructions.

**Table 1:** Strains used in this study

Strain	Characteristics	Source
*Escherichia coli* BW25113 (DE3)	Wild type with integrated DE3 lysogen	[30]
*Escherichia coli* BW25113 Δ*ackA*Δ*ldhA*	Disruption of lactate and acetate production (via *ackA*)	This study
*Escherichia coli* T7 Express	fhuA2 [lon] ompT gal (λ DE3) [dcm] ∆hsdS λ DE3 = λ sBamHIo ∆EcoRI-B int::(LacI::PlacUV5::T7 gene1) i21 ∆nin5	NEB
*Escherichia coli* NEB^®^ 5-alpha	*fhuA2 Δ(argF*-*lacZ)U169 phoA glnV44 Φ80 Δ(lacZ)M15 gyrA96 recA1 relA1 endA1 thi*-*1 hsdR17*	NEB

**Table 2:** Plasmids used in this study

Plasmid	Promoter	Gene/protein	Source
pET26b	LacI/*T7*	/	This study
pET26b:hWanEat1	LacI/*T7*	Codon harmonised *eat1* from *Wickerhamomyces anomalus* DSM 6766	[7]
pET26b:hKmaEat1	LacI/*T7*	Codon harmonised *eat1* from *Kluyveromyces marxianus* DSM 5422	This study
pET26b:hKma trEat1A-4	LacI/*T7*	Kma Eat1 truncated at A-4	This study
pET26b:hKma trEat1 P-9	LacI/*T7*	Kma Eat1 truncated at P-9	This study
pET26b:hKma trEat1 T-15	LacI/*T7*	Kma Eat1 truncated at T-15	This study
pET26b:hKma trEat1 Y-19	LacI/*T7*	Kma Eat1 truncated at Y-19	This study
pET26b:hKma trEat1 S-20	LacI/*T7*	Kma Eat1 truncated at S-20	This study
pET26b:hKma trEat1 R-24	LacI/*T7*	Kma Eat1 truncated at R-24	This study
pET26b:hKma trEat1 F-26	LacI/*T7*	Kma Eat1 truncated at F-26	This study
pET26b:hKma trEat1 N-27	LacI/*T7*	Kma Eat1 truncated at N-27	This study
pET26b:hKma trEat1 Q-28	LacI/*T7*	Kma Eat1 truncated at Q-28	This study
pET26b:hKma-trEat1-K30	LacI/*T7*	Kma Eat1 truncated at K-30	This study
pET26b:hKma-trEat1-P34	LacI/*T7*	Kma Eat1 truncated at P-34	This study
pET26b:hKma-trEat1-L35	LacI/*T7*	Kma Eat1 truncated at L-35	This study
pET26b:hKma-trEat1-P36	LacI/*T7*	Kma Eat1 truncated at P-36	This study
pET26b:hKma-trEat1-I37	LacI/*T7*	Kma Eat1 truncated at I-37	This study
pET2b:hWan-trEat1-V11	LacI/*T7*	Wan Eat1 truncated at V-11	This study
pET26b:hWan-trEat1-N13	LacI/*T7*	Wan Eat1 truncated at N-13	This study
pCas9	/		[6]
pTarget	/		[6]
pTarget-*ackA*	/		This study
pTarget-*ldhA*	/		This study

### Cultivation

*E. coli* strains were routinely grown on LB medium supplemented with kanamycin (50 μg/mL) or spectinomycin (50 μg/mL). Anaerobic experiments were performed in 250-mL serum bottles containing 50 mL modified M9 medium, which contained M9 salts (Difco, 1X), glucose (55 mM), MgSO_4_ (2 mM), CaCl_2_ * 2 H_2_O (0.1 mM), MOPS (100 mM) and 1 mL 1000X trace elements and vitamins each according to Verduyn et al. [26]. The serum bottles were made anaerobic by flushing with nitrogen gas. Precultures were made by transferring single colonies to 10 mL LB medium in a 50-mL tube and grown overnight at 30 °C and 250 rpm. The next day, 1–2 mL of the LB culture was transferred to 50 mL modified M9 medium in a 250-mL Erlenmeyer flask. The culture was grown overnight aerobically at 30 °C and 250 rpm. The anaerobic serum bottles were inoculated to an initial OD of 0.2 and incubated at 30 °C and 150 rpm. When appropriate, isopropyl β-d-1-thiogalactopyranoside—IPTG (0.01–0.5 mM) was added to induce gene expression. Experiments were performed as biological duplicates. Ethyl acetate production in serum bottles was measured only in the liquid phase.

### Visualisation of cell-free extracts by SDS-PAGE analysis

Cell lysates were prepared from frozen culture samples using B-PER (Thermo Scientific) in combination with DNase I and Lysozyme according to the manufacturer’s protocol. The soluble and insoluble fractions were separated, and the latter was washed once with 1 mL of a 10X diluted B-PER–20 mM Tris–HCl (pH 7.5) solution, and one time with 20 mM Tris–HCl (pH 7.5). The pellet was then resuspended in 20 mM Tris–HCl (pH 7.5) in a similar volume as the soluble fraction. Total protein concentrations were estimated using the DC Protein Assay (BIO RAD) according to the supplier’s manual. The soluble and insoluble fraction were diluted to equal concentrations using 20 mM Tris–HCl buffer (pH 7.5) and treated with 1X Laemmli buffer (1% SDS, 10% glycerol, 0.01% bromophenol blue, 250 mM Tris–HCl, pH 6.8, 1% beta-mercaptoethanol) for 5 min at 95 °C. A total amount of 20 µg protein sample was loaded on a BIO-RAD Criterion TGX Stain-Free Gel (4–15%), run at 200 V and stained with Bio-Safe Coomassie G-250 Stain (BIO RAD) according to manufacturer instructions.

### Protein purification

The *K. marxianus* Eat1 and its variants were purified by Strep-tag purification. *E. coli* cultures were grown aerobically in 250-mL Erlenmeyer flasks containing 100 mL modified M9 minimal medium supplemented with 100 µg/mL kanamycin. The flasks were inoculated to a starting OD_600_ of 0.05 from an overnight LB pre-culture and incubated at 25 °C and 250 rpm. After 3 h of growth, IPTG was added to a final concentration of 0.05 mM. Cultures were harvested by centrifugation at 4500×*g* and 4 °C. The pellets were resuspended in 1 mL BufferW (Strep-Tactin^®^ XT Spin Column Kit, IBA Life Sciences). The cell suspension was transferred to a tube containing Lysing matrix E (MP Biomedicals) and lysed by bead-beating for 30 s at 6500 rpm using a FastPrep^®^-24 apparatus (MP Biomedicals). The lysed cells were centrifuged for 10 min at 20,000×*g.* The supernatant was transferred to an Eppendorf tube and re-centrifuged for 10 min at 20,000×*g*. The resulting supernatant was used for protein purification. All further purification steps were performed in accordance with the Strep-Tactin^®^ XT Spin Column Purification Kit high protein yield protocol (IBA Life Sciences). The eluent containing purified protein was transferred to a Vivaspin^®^ 500,10,000 MWCO PES column (Sartorius) and concentrated by centrifugation at 15,000×*g* for 10-15 min. Protein concentration was determined with the Micro-Lowry (Onishi and Barr modification) total protein kit (Sigma-Aldrich) according to supplier instructions. A calibration curve using bovine serum albumin (BSA) was used to determine protein concentration. The *W. anomalus* Eat1 protein was purified by 6X His-tag purification as described previously [7].

### Enzyme assays

The hydrolysis of 1-naphthyl acetate (1-NA) was measured spectrophotometrically by monitoring the release of 1-naphthol at 320 nm [5] in a Synergy MX temperature regulated plate reader (BioTek) at the desired temperature. Assays were performed in 96-well flat-bottom plates (Greiner) in a final volume of 100 µL. The well contained sodium phosphate buffer (50 mM, pH 7.5), NaCl (100 mM) and 1-naphthyl acetate (0.5 mM). The reaction was initiated by adding purified protein to a final concentration of 10 µg/mL. Residual esterase activity assays were performed by incubating the enzymes in sodium phosphate buffer (50 mM, pH 7.5) containing NaCl (100 mM) in a PCR thermocycler at the desired temperature. Aliquots were taken at various incubation times (0–90 min) and assayed for 1-naphthyl acetate hydrolysis at 40 °C. A calibration curve was used to calculate the concentration of 1-naphthol released in the reaction. Specific activity was defined as the amount of protein (mg) required to form 1 µmol of 1-naphthol, per min. Measurements were performed as technical triplicates.

### Bioinformatics

Mitochondrial pre-sequences and amino acid (AA) positions of typical cleavage sites were identified using the prediction tool MitoFates [4]. The translation initiation rates of ribosome binding sites were predicted with the RBS Calculator v2.1. The predictions were performed for *E. coli* MG1655 (ACCTCCTTA).

### Analytical

Glucose and organic acids were analysed by high-performance liquid chromatography (HPLC) on an Agilent 1290 LC II system, equipped with an Agilent 1290 Infinity Binary Pump, Agilent 1290 Infinity Autosampler, Agilent 1290 Infinity diode array detector operated at 210 nm, and an Agilent 1260 Infinity RI detector operated at 45 °C. Either an Aminex HPX-87H (Bio-Rad) or an Rezex ROA-Organic Acid H + (Phenomenex) column was used with a mobile phase of 0.008 mM H_2_SO_4_. The HPLC was operated at 0.8 mL/min and 60 °C. Propionic acid (50 mM) was used as internal standard.

Ethyl acetate and ethanol in liquid samples were measured by an Agilent 7890B gas chromatograph equipped with a flame ionisation detector (GC-FID) and an Agilent 7693 autosampler. Samples were analysed by injecting 0.5 μL of liquid sample onto a Nukol™ column (30 m × 0.53 mm, 1.0 μm coating, Supelco). The column temperature was maintained at 50 °C for 2 min and increased to 200 °C at a rate of 50 °C/min. The split ratio was 10. 1-Butanol (2 mM) was used as the internal standard.

## Supplementary information

**Additional file 1: Figure S1.** SDS-PAGE analysis of CFE of *E. coli* cultures producing various Eat1 variants. Soluble and insoluble fractions of CFE were prepared from cultures induced with 0.01 mM IPTG after 70 h of anaerobic cultivation. Two biological replicates (A and B) of each culture were analysed. Uninduced cultures were used as a control. The Precision Plus Protein Kaleidoscope Standard (BIO RAD) was added in lanes 1 and 18. Kma Eat1 variants are shown in (a) and Wan Eat1 variants are shown in (b). The Eat1 proteins are expected at a band size of approximately 42 kDa. Truncation of Eat1 had only a minor effect on the size of the protein, whichwas not detectable by SDS-PAGE.

**Additional file 2: Figure S2.** Lack of correlation between ethyl acetate formation and predicted strength of the RBS controlling the production of *K. marxianus* trEat1. Ethyl acetate titres were obtained from *E. coli* BW25113 Δ*ackA*Δ*ldhA* (DE3) producing *K. marxianus* trEat1 variants at 0.01 mM IPTG concentration (Fig. 2b). The translation initiation rates of the RBS were predicted with the RBS calculator [22].

**Additional file 3: Figure S3.** Thermal inactivation measurements used to determine the inactivation constants (*k*_*i*_) of three *K. marxianus* Eat1 variants at 45 °C, 50 °C and 55 °C.

## Data Availability

All data generated or analysed during this study are included in this published article and its additional information files.

## Abbreviations

1-NA: 1-Naphthyl acetate
AA: Amino acid
AAT: Alcohol acetyl transferase
ackA: Acetate kinase
Atf1: Alcohol acetyltransferase 1
Cfa: *Cyberlindnera fabianii*
Cja: *Cyberlindnera jadinii*
Eat1: Ethanol acetyltransferase 1
Ecy: *Eremothecium cymbalariae*
Huv: *Hanseniaspora uvarum*
Icp55: Intermediate cleaving peptidase 55
IPTG: Isopropyl β-d-1-thiogalactopyranoside
Kla: *Kluyveromyces lactis*
Kma: *Kluyveromyces marxianus*
Ldh: Lactate dehydrogenase
MPP: Mitochondrial processing peptidase
Oct1: Octapeptidylpeptidase 1
Sce: *Saccharomyces cerevisiae*
TOM: Complex Translocase of the outer mitochondrial membrane complex
trEat1: Truncated Eat1
upEat1: Unprocessed Eat1
Wan: *Wickerhamomyces anomalus*
Wci: *Wickerhamomyces ciferrii*
