# Supplementary figures and images for: From Eat to trEat: engineering the mitochondrial Eat1 enzyme for enhanced ethyl acetate production in Escherichia coli
Source: Biotechnol Biofuels. 2020 Apr 19;13:76. doi: 10.1186/s13068-020-01711-1 (PMC7168974; doi:10.1186/s13068-020-01711-1)

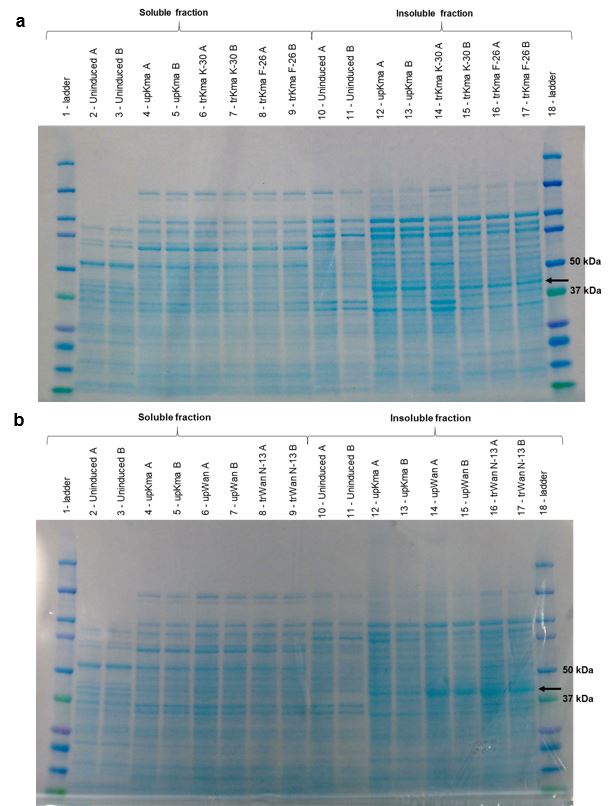

Supplement: Supplementary file 1 — Additional file 1: Figure S1. SDS-PAGE analysis of CFE of E. coli cultures producing various Eat1 variants. Soluble and insoluble fractions of CFE were prepared from cultures induced with 0.01 mM IPTG after 70 h of anaerobic cultivation. Two biological replicates (A and B) of each culture were analysed. Uninduced cultures were used as a control. The Precision Plus Protein Kaleidoscope Standard (BIO RAD) was added in lanes 1 and 18. Kma Eat1 variants are shown in (a) and Wan Eat1 variants are shown in (b). The Eat1 proteins are expected at a band size of approximately 42 kDa. Truncation of Eat1 had only a minor effect on the size of the protein, whichwas not detectable by SDS-PAGE. [file 13068_2020_1711_MOESM1_ESM.jpg]

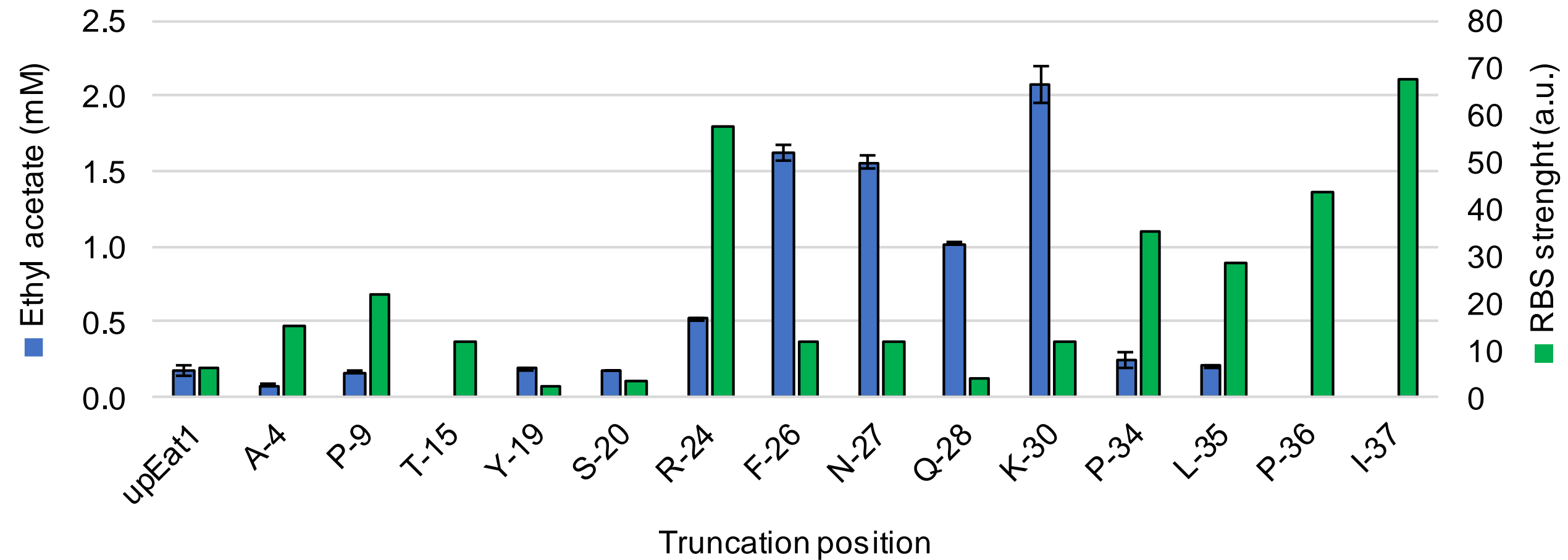

Supplement: Supplementary file 2 — Additional file 2: Figure S2. Lack of correlation between ethyl acetate formation and predicted strength of the RBS controlling the production of K. marxianus trEat1. Ethyl acetate titres were obtained from E. coli BW25113 ΔackAΔldhA (DE3) producing K. marxianus trEat1 variants at 0.01 mM IPTG concentration (Fig. 2b). The translation initiation rates of the RBS were predicted with the RBS calculator [22]. [file 13068_2020_1711_MOESM2_ESM.pdf]

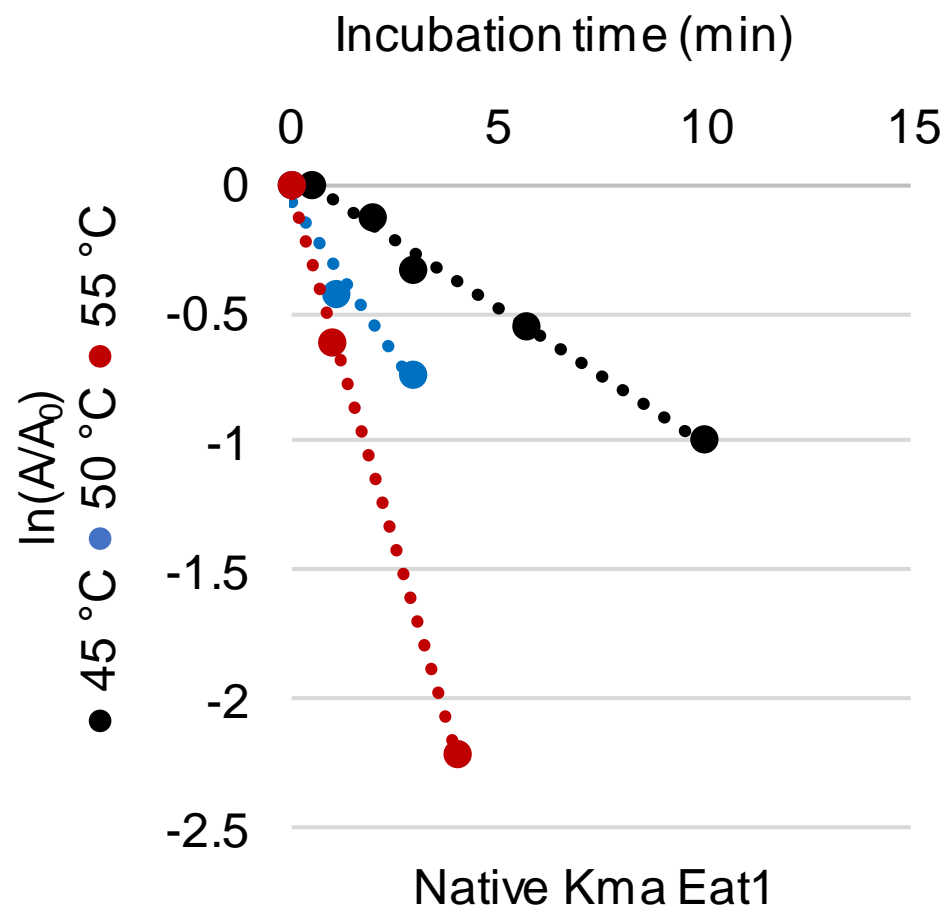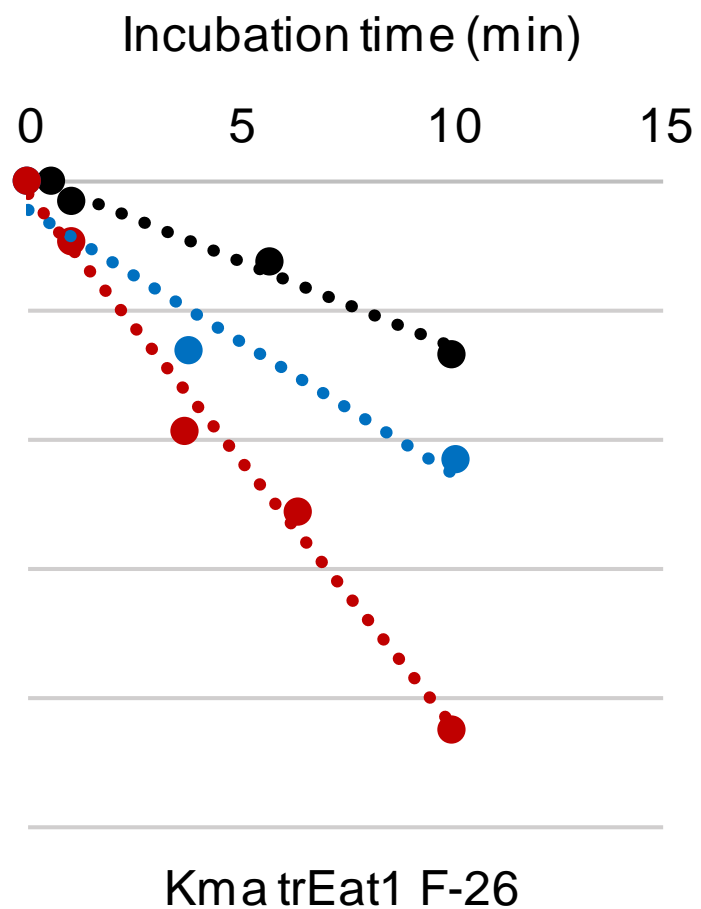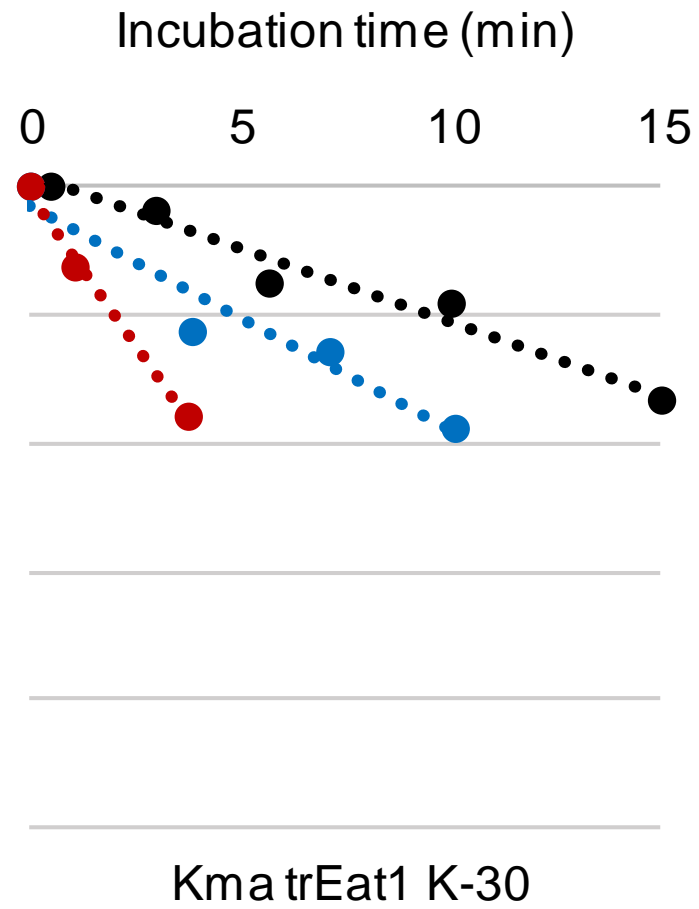

Supplement: Supplementary file 3 — Additional file 3: Figure S3. Thermal inactivation measurements used to determine the inactivation constants (ki) of three K. marxianus Eat1 variants at 45 °C, 50 °C and 55 °C. [file 13068_2020_1711_MOESM3_ESM.pdf]
